# THE MENTAL HEALTH BENEFIT OF FRIEND NETWORKS IN OLDER KOREAN AMERICANS: THE CONDITIONING EFFECT OF FAMILY TYPE

**DOI:** 10.1093/geroni/igac059.2637

**Published:** 2022-12-20

**Authors:** Yuri Jang, Nan Sook Park, Juyoung Park, David Chiriboga, William Haley, Miyong Kim

**Affiliations:** University of Southern California, Los Angeles, California, United States; University of South Florida, Tampa, Florida, United States; University of Southern California, Los Angeles, California, United States; University of South Florida, Tampa, Florida, United States; University of South Florida, Tampa, Florida, United States; University of Texas at Austin, Austin, Texas, United States

## Abstract

Building on the importance of family and friends as sources of social connectedness in later years of life, we conducted a contextual examination of their independent and interactive roles in predicting mental health, using a compensatory social convoy model. In a sample of older Korean Americans, we anticipated that friend networks would be a more important predictor of mental distress when strong family relationships were absent. Data were from 2,140 participants in the Study of Older Korean Americans, a multi-state survey of Korean immigrants age 60 and older (Mage = 73.4, SD = 7.97). To identify family types, latent profile analysis (LPA) was performed with 17 variables assessing family networks, positive and negative interactions with family members, and incidence of mistreatment by family. Linear regression models of mental distress then examined the direct effects of family type and networks of friends, as well as their interactions. LPA on family-related items identified three family types: close-knit, mixed, and dysfunctional. Membership in the close-knit group and a larger network of friends were associated with lower levels of mental distress. In addition, a significant interaction was found between dysfunctional family type and friend network (B = −.35, SE = .08, p < .001), where the positive effect of friend networks was most pronounced in the context of dysfunctional family relationships. These findings support the use of a compensatory social convoy model. Substituting for family resources, friend networks yielded compensatory mental health protection for those with dysfunctional family relations.

